# Knowledge and Adherence to Lifestyle Habits to Prevent Complications Associated With Immunosuppression in Kidney Transplant Recipients: A Single‐Center Survey

**DOI:** 10.1111/tid.70038

**Published:** 2025-04-26

**Authors:** Tamara Ruiz‐Merlo, Isabel Rodríguez‐Goncer, Francisco López‐Medrano, Natalia Polanco, Esther González, Hernando Trujillo, Marina Fayos, Natalia Redondo, Rafael San Juan, Amado Andrés, José María Aguado, Mario Fernández‐Ruiz

**Affiliations:** ^1^ Unit of Infectious Diseases, Hospital Universitario “12 de Octubre,” Instituto de Investigación Sanitaria Hospital “12 de Octubre” (imas12) Madrid Spain; ^2^ Centro de Investigación Biomédica en Red de Enfermedades Infecciosas (CIBERINFEC), Instituto de Salud Carlos III Madrid Spain; ^3^ Department of Medicine School of Medicine, Universidad Complutense Madrid Spain; ^4^ Department of Nephrology Hospital Universitario '12 de Octubre', Instituto de Investigación Sanitaria Hospital '12 de Octubre' (imas12) Madrid Spain

**Keywords:** food safety, kidney transplantation, lifestyle habits, self‐reported vaccination, survey

## Abstract

**Background:**

Chronic immunosuppression associated with certain lifestyle habits render kidney transplant (KT) recipients more susceptible to infection and cancer. We assessed the level of knowledge and adherence to safe living strategies to minimize the occurrence of posttransplant complications.

**Methods:**

Consecutive KT recipients were offered a self‐administered questionnaire covering the following areas: demographics and socioeconomic factors; generic hygiene habits; sun exposure; smoking and alcohol consumption; vaccination status; animal contact and gardening; international travelling; and food safety and habits.

**Results:**

Between May 2019 and May 2021, 130 KT recipients responded the survey at a median of 61.5 posttransplant days (completion rate of 94.9%). Only 19.7% of participants visited the dentist at least every 3–6 months. Although the majority (88.5%) were aware of the need of sunscreen, only 23.3% used it throughout the year. Self‐reported influenza vaccine uptake in the last session was 69.1%. Pet ownership was reported by 41.7% of participants, of which more than one‐third had considered to give up the care of their animals. Gardening and international travel were uncommon. A notable proportion of participants acknowledged to consume the following products either “usually” or “often”: raw or undercooked meat (12.4%), undercooked fish (24.8%), raw seafood (8.8%), homemade sausages or cured ham (51.5%), pâté or meat spreads (35.2%), and “ready‐to‐eat” salads (31.8%). Adherence was poorer among non‐native‐speaking patients and those with lower education and household incomes.

**Conclusion:**

There is room for improvement in health education and promotion practices among KT recipients, particularly those with potential cultural and socioeconomic barriers.

## Graphical abstract

We performed a survey on lifestyle habits aimed at reducing the risks associated with immunosuppression in kidney transplant recipients. We found gaps in dental hygiene, sunscreen use, vaccination, and consumption of high‐risk foods. Cultural and economic factors influenced adherence. Health education could minimize posttransplant complications by promoting safe living strategies.

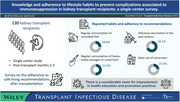

**Abbreviations**
AST‐IDCOP: American Society of Transplantation Infectious Diseases Community of Practice
ESRD: end‐stage renal disease
HAV: hepatitis A virus
HEP: health education and promotion
HEV: hepatitis E virus
HRQoL: health‐related quality of life
IQR: interquartile range
ISCED: International Standard Classification of Education
KT: kidney transplantation
MMR: measles, mumps, and rubella
SD: standard deviation
SOT: solid organ transplantation

## 1 Introduction

Kidney transplantation (KT) represents the best treatment option for patients with end‐stage renal disease (ESRD). In comparison to alternative renal replacement therapies, KT has been proven to reduce mortality [1, 2] and to improve health‐related quality of life (HRQoL) [3]. The specific characteristics of KT recipients, however, put them at increased risk of developing graft loss and serious complications, ultimately requiring lifelong follow‐up.

The level of adherence to immunosuppressive therapy throughout the patient's lifespan is recognized as a relevant factor to minimize the occurrence of graft rejection and loss [4]. On the other hand, long‐term immunosuppression in association with certain high‐risk lifestyles render KT recipients more susceptible to the development of infection, cancer, and cardiovascular disease, in particular once the patients resume their occupational and recreational activities. Cancer‐related mortality is two to three times higher in solid organ transplant (SOT) recipients as compared to the general population [5, 6]. Although many of the factors predisposing to posttransplant cancer are nonmodifiable (e.g. older age or latent viral infections), the compliance with lifestyle modifications regarding sun exposure or smoking habits contributes to risk minimization [7]. Similarly, dietary habits, pet ownership practices, or environmental exposures in the setting of immunosuppression influence the risk of foodborne infections, zoonosis, or fungal diseases [8, 9]. In addition, there remains room for improvement in terms of vaccine‐preventable infections in the SOT population [10, 11].

Health education and promotion (HEP) is crucial to provide patients with accessible and evidence‐based recommendations on lifestyle habits. This process does not only consist of transmitting information, but also aims at actively empowering patients through a structured program that fosters the acquisition of knowledge, skills, and self‐care capacities [12]. In their condition of chronic patients that must receive a treatment that is not without potentially serious adverse events, KT recipients constitute a preferential target for HEP [13, 14, 15].

To guide any HEP intervention program in the SOT population, it is first necessary to assess the awareness and compliance with safe living strategies, as well as to identify potential attitudinal and educational barriers. Previous studies on this issue, however, are scarce [8, 16, 17]. Therefore, we conducted a survey among KT recipients to describe the level of knowledge and adherence to common recommendations aimed at preventing the occurrence of complications associated with immunosuppression, such as community‐acquired infections and de novo cancer, and the nature of the lifestyle modifications eventually implemented upon transplantation.

## 2 Methods

### 2.1 Study Population and Design

The present survey was conducted in adult patients (≥ 18 years) that consecutively underwent KT at the Hospital Universitario “12 de Octubre” (Madrid, Spain) between May 2019 and May 2021. The research was performed in accordance with the ethical standards laid down in the Declarations of Helsinki and Istanbul and the applicable Spanish legislation. The study protocol was approved by the local Clinical Research Ethics Committee (CEIC no. 19/340).

Outpatient KT recipients with a functional graft under immunosuppressive therapy were offered to participate in the survey at posttransplant Months 1–3. The survey was based on a self‐administered questionnaire that was completed in person on a single occasion. Since the questionnaire was designed in Spanish only, additional measures were implemented to ensure the correct understanding of the information in patients with limited Spanish proficiency. These participants were offered a detailed explanation of each question with the assistance of the patient's companion or relative, and we subsequently verified that the content had become clear. In addition, we used the services of or a professional translator if necessary. In case of sensory deficits, the patient may be assisted by a family member, but researchers assured that only his/her own opinion was captured. Patients with moderate‐to‐severe cognitive impairment or unstable clinical condition at the time of recruitment were excluded.

Approached patients were informed on the purpose of the study and the voluntary and anonymous nature of the survey. They were asked to ensure that their responses reflected their actual perceptions and practices as accurately as possible. In addition, they were invited to share any doubt that they might have regarding their recent condition as transplant recipients. Those that expressed interest in participating were provided with an informed consent and the questionnaire. No remuneration was offered for completing the survey.

### 2.2 Study Procedure and Definitions

No formal education on safe living recommendations after transplantation was systematically offered to patients on the waiting list in our institution. Once in the hospitalization ward following transplant surgery, KT recipients were routinely given in an unstructured manner recommendations regarding adherence to immunosuppressive drugs and antimicrobial prophylaxis by the medical and nursing staff. This approach did not guarantee that all patients received identical information or were provided with a written guideline (i.e. continuity of nursing care report) associated with their hospital discharge report.

The survey was designed to assess the level of knowledge and adherence to a set of recommendations aimed at minimizing the occurrence of posttransplant complications (namely infections and cancer) in recent KT recipients. The content of these recommendations was aligned with the American Society of Transplantation Infectious Diseases Community of Practice (AST‐IDCOP) guidelines on safe living strategies following SOT [18]. The survey contained a total of 79 questions that were answered as “yes” or “no,” on a Likert scale (five frequency response options), or in a free text format. The standardized questionnaire was divided into the following domains: (a) demographics and socioeconomic factors (e.g. age, gender, country of birth, maximum educational level, marital status, current labor status, and household composition); (b) generic hygiene habits (e.g. hand washing and tooth brushing); (c) sun exposure habits; (d) smoking and alcohol consumption; (e) vaccination status; (f) contact with animals and gardening (e.g. pet ownership); (g) international travelling; and (g) food safety and habits (e.g. consumption of raw or undercooked meat or seafood, “ready‐to‐eat” salads or unpasteurized milk derivatives). The questionnaire was tested for clarity and comprehensibility in the first five recruited patients and subsequently amended if necessary.

Data on pretransplant comorbidities, underlying ESRD, donor characteristics, perioperative variables, immunosuppression therapy, and posttransplant complications were prospectively collected by means of a standardized case report form within the institutional cohort [19].

The maximum educational level achieved by the participant was categorized according to the 2011 International Standard Classification of Education (ISCED) [20]. Data on the average household income were obtained from the Household Income Distribution Atlas (*Atlas de Distribución de Renta de los Hogares*) corresponding to the year 2022, which is provided by the Spanish National Statistics Institute (*Instituto Nacional de Estadística*). This tool is based on the exploitation of administrative registers from national and regional tax agencies and offers granular data on the level and distribution of household incomes of the Spanish population, broken down by territorial levels (i.e. census sections, districts, municipalities, provinces, and autonomous communities). For a better comparison across different types of households, the equivalent income is standardized by the number of equivalent consumption units that make up each household [21]. We collected the average income data available for the smallest territorial unit corresponding to the usual address of residence of the participant.

### 2.3 Statistical Analysis

Descriptive statistics were used to summarize participant characteristics and survey responses. Some responses were collapsed for analysis purposes. The sample size (i.e. denominator) for each question did not include missing or “not applicable” responses. Quantitative data were shown as the mean ± standard deviation (SD) or the median with interquartile range (IQR), whereas qualitative variables were expressed with absolute and relative frequencies. Categorical variables were compared with the *χ* ^2^ test or Fisher's exact test. Average household incomes were categorized by tertiles based on the distribution within the study population. The association between various sociodemographic and clinical characteristics and the degree of awareness and adherence to the recommendations was investigated. In detail, we assessed the impact of patient age, gender, membership of a patients' association, being a non‐native Spanish speaker, ISCED level, history of previous SOT, and the annual household income. To this aim, “usually” or “often” responses in questions on dietary habits were grouped. Statistical analysis was performed using SPSS version 20.0 (IBM Corp., Armonk, NY).

## 3 Results

### 3.1 Characteristics of the Study Cohort

Overall, 137 consecutive KT recipients during the study period were approached, of which 130 (94.9%) agreed to participate in the survey and completed the questionnaire at a median of 61.5 days (IQR: 42.8–95.3) after transplantation. Demographics and clinical and socioeconomic characteristics of this cohort are detailed in Table 1.

**TABLE 1:** Sociodemographic characteristics of the study cohort (*n* = 130).

Variable	
Age, years [mean ± SD]	55.6 ± 15.2
Gender (male) [*n* (%)]	83 (63.8)
Country of origin [*n* (%)]	
Spain	108 (83.1)
Latin America^a^	16 (12.3)
Eastern Europe^b^	4 (3.1)
Other^c^	2 (1.5)
Non‐native Spanish speaker [*n* (%)]	8 (6.2)
Marital status [*n* (%)]	
Married	66 (50.8)
Single	43 (33.1)
Separated or divorced	12 (9.2)
Widowed	9 (6.9)
Maximum educational level [*n* (%)]	
No formal education or incomplete primary education	25 (19.2)
Primary education	13 (10.0)
Lower secondary education (general or vocational)	29 (22.3)
Upper secondary education (general or vocational)	34 (26.2)
University studies	29 (22.3)
Current labor status [*n* (%)]^d^	
Active, currently working	65 (50.8)
Retired	31 (24.2)
Temporary or permanent sick leave	12 (9.4)
Active, unemployed	11 (8.5)
Housekeeper	7 (5.5)
Other	2 (1.6)
Household composition [*n* (%)]	
Live with spouse or partner	51 (39.2)
Live with spouse or partner and children	19 (14.6)
Live alone	13 (10.0)
Live with children	11 (8.5)
Live with parents	10 (7.7)
Other	26 (20.0)
Membership of a patients' association [*n* (%)]	18 (15.5)
Annual average income per household, euros [median (IQR)]	32 995.6 (29 307.4–40 928.6)

Abbreviations: IQR: interquartile range; SD: standard deviation.

^a^ Venezuela (*n* = 5), Ecuador (*n* = 3), Colombia (*n* = 2), Honduras (*n* = 2), Brazil (*n* = 2), Peru (*n* = 1), and Bolivia (*n* = 1).

^b^ Romania (*n* = 2), Poland (*n* = 1), and Bulgaria (*n* = 1).

^c^ Morocco (*n* = 1) and Ecuatorial Guinea (*n* = 1).

^d^ Data on labor status was not provided by two patients.

The mean age of participants was 53.5 ± 15.2 years. Most of them were males (63.1%), Spaniards (83.1%), married (50.8%), and lived with their spouse or partner (39.2%). Regarding the ISCED educational level, half (48.5%) reported to have upper secondary education or university studies. Most patients (50.8%) were working at the time of survey. Membership of a patients' association (typically for chronic kidney diseases) was reported by 15.5%.

Regarding clinical characteristics, a majority of patients (89.2%) had received renal replacement therapy before transplantation for a median duration of 2.9 years. Diabetic nephropathy (19.2%) and glomerulonephritis (16.9%) were the most common causes of ESRD. About one quarter (23.8%) had undergone previous SOT (Table 2).

**TABLE 2:** Clinical characteristics of the study cohort (*n* = 130).

**Variable**	
Pretransplant comorbidities [*n* (%)]	
Diabetes mellitus	
Hypertension	101 (77.7)
Dyslipidemia	66 (50.8)
Diabetes mellitus	36 (27.7)
Cerebrovascular disease	4 (3.1)
Coronary artery disease	16 (12.3)
Chronic respiratory disease	12 (89.2)
Type of transplantation [*n* (%)]	
Single kidney	118 (90.8)
Pancreas–kidney	9 (6.9)
Liver–kidney	2 (1.5)
Heart–kidney	1 (0.8
Etiology of ESRD [*n* (%)]	
Diabetic nephropathy	25 (19.2)
Glomerulonephritis	22 (16.9)
Polycystic kidney disease	19 (14.6)
Chronic interstitial kidney disease	11 (8.5)
Nephroangiosclerosis	10 (7.7)
Vasculitis‐associated and lupus nephropathy	6 (4.6)
Congenital nephropathy	6 (4.6)
Reflux nephropathy	6 (4.6)
Other	9 (6.9)
Unknown	16 (12.3)
Previous solid organ transplantation [*n* (%)]	31 (23.8)
≥ 2 previous grafts	6 (4.6)
Type of previous graft^a^	
Kidney	39/42 (92.9)
Pancreas–kidney	2/42 (4.8)
Liver	1/42 (2.4)
Previous renal replacement therapy [*n* (%)]	116 (89.2)
Hemodialysis	88/116 (75.9)
Peritoneal dialysis	28/116 (24.1)
Time on dialysis, days [median (IQR)]	1078 (650–1693)
Type of donor [*n* (%)]	
Living donor	25 (19.2)
Donation after brain death donor	92 (70.8)
Donation after circulatory death donor	13 (10.0)
Induction therapy [*n* (%)]	
Antithymocyte globulin	64 (56.1)
Basiliximab	50 (43.9)
None	16 (12.3)
Primary immunosuppression regimen [*n* (%)]	
Prednisone, tacrolimus, and MMF/MPS	119 (91.5)
Prednisone, tacrolimus, and azathioprine	9 (6.9)
Prednisone, tacrolimus, and everolimus	2 (1.5)

Abbreviations: ATG: antithymocyte globulin; ESRD: end‐stage renal disease; IQR: interquartile range; MMF/MPA: mycophenolate mofetil / mycophenolic acid; SD: standard deviation.

^a^ On the total number of previous grafts.

### 3.2 Generic Hygiene and Health Recommendations and Vaccination

Most participants said that they never visited the podiatrist or chiropodist (66.1%), whereas only less than one‐fifth (18.1%) visited the dentist at least every 3–6 months. Almost half (48.4%) brushed their teeth twice a day or less often. About one‐third (37.2%) of the participants that usually depilated reported to use a razor. Although the majority (88.5%) were aware of the recommendations to use sunscreen, less than a quarter (23.3%) reported to apply it throughout the year. Regarding vaccination practices, self‐reported influenza vaccine uptake in the last or previous seasons were 69.1% and 67.2%, respectively (Table 3). Although the questionnaire included a set of questions about other types of vaccines (pneumococcal, hepatitis A and B, tetanus, and measles, mumps, and rubella [MMR]), non‐response rates were very high for all of them (ranging from 42.3% to 84.6%), preventing further analysis.

**TABLE 3:** Adherence to generic hygiene and health recommendations and vaccination.

Question	Response [*n* (%)]
Do you regularly check your feet, paying attention to and keeping dry the toe creases in particular?	108 (83.1)
Do you get a manicure or pedicure in beauty salons?	22 (17.9)
How often do you visit the podiatrist or chiropodist?	
Once a month	10 (7.9)
Every 3–6 months	13 (10.2)
Every year	20 (15.7)
Never	84 (66.1)
How often do you visit the dentist?	
Once a month	2 (1.6)
Every 3–6 months	23 (18.1)
Every year	88 (69.3)
Never	14 (11.0)
How often do you wash your hands?	
Before eating	123 (96.9)
Once a day	2 (1.6)
I do not usually wash my hands	2 (1.6)
What type of soap do you use?	
Liquid soap	102 (81.6)
Soap bar	18 (14.4)
Both	5 (4.0)
How often do you brush your teeth?	
After meals	66 (51.6)
Once or twice a day	59 (46.1)
Never	3 (2.3)
How often do you renew your toothbrush?	
Once a year	15 (12.0)
Every 3–6 months	92 (73.6)
Only if very deteriorated	18 (14.4)
Do you depilate?	51 (40.2)
If yes, what depilation method do you use?	
Razor shaving	16 (37.2)
Depilatory cream	7 (16.3)
Waxing	6 (14.0)
Electric shaver	5 (11.6)
Laser or pulsed light	4 (9.3)
How often do you take a shower or bath?	
Daily	106 (81.5)
Every 2 days	17 (13.1)
Twice a week	6 (4.6)
Once a week	1 (0.8)
Are your toiletries (e.g. toothbrush, razors, nail clippers, etc.) for your exclusive use?	128 (98.5)
Did you know that you should avoid contact with people with fever or symptoms of ongoing infection?	125 (96.9)
Do you know that you should use sunscreen?	115 (88.5)
When do you use sunscreen?	
Daily during all the year	30 (23.3)
Only during summer months	53 (41.1)
Only when I expose myself (e.g. on the beach)	38 (29.5)
Never	8 (6.2)
Do you smoke, or have you ever smoked regularly?	
I've never smoked	53 (40.8)
I used to smoke, but not anymore	71 (54.6)
I currently smoke	6 (4.6)
How often do you drink alcohol or have a drink containing alcohol?	
Daily	1 (0.8)
Once a week	6 (4.6)
Once a month	3 (2.3)
Very sporadically	36 (27.7)
Never	83 (64.3)
Do you do sports regularly?	44 (34.6)
Did you get the influenza vaccine last year?	85 (69.1)
Have you been vaccinated against influenza in previous years?	78 (67.2)

### 3.3 Recommendations on Animal Contact, Gardening, and International Travelling

The rate of participants that reported owning a pet, typically a dog, was high (41.7%). All of them stated that their vaccination and deworming schedules were up to date. In most cases it was someone else who used to be involved in taking care of (50.9%) and washing the animal (79.6%). More than one‐third of patients (37.5%) had considered to give up the care of their pet following transplantation. Gardening and international travelling during the previous year were rarely reported (17.8% and 5.5%, respectively). Only three out of seven patients (60.0%) had informed their nephrologist in advance of their plans to travel (Table 4).

**TABLE 4:** Adherence to recommendations on animal contact, gardening, and travelling.

Question	Response [*n* (%)]
Do you own a pet?	53 (41.7)
If yes, how long have you had the animal (years)? [median (IQR)]	8.0 (4.0–11.3)
Type of pet^a^	
Dog	43 (81.1)
Cat	12 (22.6)
Bird	4 (7.5)
Other	1 (1.9)
Are your pet's vaccinations and deworming schedule up to date?	53 (100.0)
When did you last take your pet to the vet (months)? [median (IQR)]	1.5 (1.0–4.0)
Who usually takes care of the animal?	
Myself	17 (32.1)
Another person	27 (50.9)
Myself and another person	9 (17.0)
Do you usually wash your hands after petting an animal?	48 (90.6)
Who usually washes the animal?	
Myself	6 (12.2)
Another person	39 (79.6)
Myself and another person	4 (8.2)
Do you usually pick up the animal's feces and clean its trough?	16 (30.2)
Have you ever considered to hand over the usual care of your pet once you received the transplant?	18 (37.5)
Are you a livestock farmer or do you have usual contact with farm animals?	2 (3.8)
Do you usually grow a garden or work an allotment?	23 (17.8)
Do you use working or gardening tools?	21 (16.2)
Did you travel abroad in the last year?	7 (5.5)
If yes, did you previously consult your nephrologist about your intention to travel?	3 (60.0)
Are you planning to travel abroad in the near future?	26 (20.6)

^a^ Multiple responses were possible.

### 3.4 Recommendations on Food Safety and Habits

A relevant proportion of participants stated to consume the following products either “usually” or “often”: raw or undercooked meat (12.4%), undercooked fish (24.8%), raw seafood (8.8%), homemade sausages or cured ham (51.5%), pâté or meat spreads (35.2%), unpasteurized cheese (27.7%), and “ready‐to‐eat” salads (31.8%). In addition, only half of them (52.0%) used to wash the packaged salads before consumption. Purchasing cheese from farmhouses or artisan cheese dairies was not exceptional (7.8%). The consumption of foods after the “best‐before” date was acknowledged to be carried out “usually” or “often” by 1.6% and 7.0%, respectively (Table 5).

**TABLE 5:** Adherence to recommendations on food safety and habits.

Question	Response [*n* (%)]
Do you eat raw or undercooked meat (e.g. *carpaccio*, steak tartar, smoked meat, etc.)?	
Usually	1 (0.8)
Often	15 (11.6)
Occasionally	10 (7.8)
Never	103 (79.8)
Do you eat raw or undercooked fish (e.g. pickled anchovies, sushi, smoked fish, etc.)?	
Usually	1 (0.8)
Often	31 (24.0)
Occasionally	19 (14.7)
Never	78 (60.5)
Do you eat raw seafood or shellfish?	
Usually	0 (0.0)
Often	11 (8.8)
Occasionally	19 (15.2)
Never	95 (76.0)
Do you eat homemade sausages or cured ham?	
Usually	9 (7.0)
Often	57 (44.5)
Occasionally	30 (23.4)
Never	32 (25.0)
Do you eat pâté or meat spreads?	
Usually	2 (1.6)
Often	43 (33.6)
Occasionally	18 (14.1)
Never	65 (50.8)
Do you usually eat goat cheese?	48 (70.6)
Do you usually eat unpasteurized cheese (e.g. roquefort, brie, camembert, and blue cheese)?	36 (27.7)
Where do you usually buy cheese?^a^	
Shops or supermarkets	99 (96.1)
Farmhouses or artisan cheese dairies	8 (7.8)
Do you consume pasteurized milk or dairy products?	
Usually	77 (60.6)
Often	23 (18.1)
Occasionally	13 (10.2)
Never	14 (11.0)
Do you wash your fruit and vegetables with bleach (sodium hypochlorite) before eating them?	70 (55.1)
Do you eat prepared and packaged “ready‐to‐eat” salads?	
Usually	8 (6.2)
Often	33 (25.6)
Occasionally	28 (21.7)
Never	60 (46.5)
Do you wash these salads before eating them?	
Usually	51 (52.0)
Often	7 (7.1)
Occasionally	7 (7.1)
Never	33 (33.7)
Do you pay attention to the condition of the eggs before eating them?	115 (89.8)
Do you usually add salt to your food?	
When do you add salt?	24 (18.8)
During cooking	58 (84.1)
At the table, before tasting the food	11 (15.9)
Do you usually consume food after the “best‐before” date?	
Usually	2 (1.6)
Often	9 (7.0)
Occasionally	9 (7.0)
Never	108 (84.4)

^a^ Multiple responses were possible.

Three‐quarter of the participants (74.6%) answered positively when they were asked if they had been previously informed of the aforementioned recommendations. The most common sources of information were the patient's nephrologist (74 [58.7%]), nurse (19 [15.1%]) or primary care physician (12 [9.5%]).

### 3.5 Sociodemographic Determinants of Knowledge and Adherence

Finally, the potential impact of sociodemographic and clinical features on the degree of awareness and adherence was assessed. Patients ≥ 70 years were more likely to report that they visit the podiatrist at least every year as compared to those below (70.0% [14/20] vs. 27.1% [29/107], respectively; *p* value < 0.001). Non‐native Spanish‐speaking recipients were less likely to brush their teeth after every meal (12.5% [1/8] vs. 54.2% [65/120]; *p* value = 0.029). This group reported more frequently to eat raw or undercooked meat (37.5% [3/8] vs. 10.7% [13/121]; *p* value = 0.060) or seafood (37.5% [3/8] vs. 6.8% [8/117]; *p* value = 0.023). Participants that had received primary education or less were less likely to consume raw or undercooked fish (10.8% [4/37] vs. 30.4% [28/92]; *p* value = 0.020) or unpasteurized cheese (13.2% [5/38] vs. 33.7% [31/92]; *p* value = 0.017) as compared to more educated participants. Patients within the lowest tertile of the household income reported to visit the dentist only “once a year” or “never” in a higher proportion than those within the intermediate or highest tertiles (9.5% [4/42] vs. 24.7% [21/85]; *p* value = 0.043). The reported adherence to daily sunscreen (11.9% [5/42] vs. 28.7% [25/87]; *p* value = 0.034) was also lower in patients in the lowest income tertile. On the contrary, patients in the highest tertile reported more frequently to consume unpasteurized cheese (39.1% [17/43] vs. 21.8% [19/87]; *p* value = 0.034) and pasteurized milk or dairy products (88.4% [38/43] vs. 73.8% [62/84]; *p* value = 0.058) as compared to lower tertiles.

No significant associations were observed for the remaining factors explored (data not shown).

## 4 Discussion

The present study provides an insight into the level of knowledge and adherence to common recommendations to minimize the risk of infection and other complications attributable to immunosuppression in an unselected, consecutive cohort of KT recipients. Our results reveal a considerable room for improvement—in particular in practices involving foot safety, oral hygiene, and sun protection—and highlight the need for specific HEP interventions [13, 14, 15]. The effective promotion of positive changes in health behaviors during the early posttransplant adaptation process should take into account the socioeconomic barriers identified for recipients with cultural diversity and lower education and income levels.

A relevant finding is the overall low rate of awareness regarding dietary habits that increase the risk of potentially life‐threatening food‐borne infections in immunocompromised patients, such as listeriosis [22], salmonellosis [23], campylobacteriosis [24], toxoplasmosis [25], and hepatitis A or E [26, 27]. Self‐reported rates of high‐risk food practices were notable for homemade sausages or cured ham, pâté or meat spreads, and “ready‐to‐eat” salads, which were reported to be consumed at least “often” by 31.8%–51.5% of participants. A survey performed among Swiss SOT recipients also revealed a low degree of adherence to food‐safety recommendations, particularly for the avoidance of raw charcuterie and meat cooked rare [8]. In contrast, about 85% of Canadian lung transplant recipients stated to avoid raw or uncooked meat or poultry [16]. These differences may reflect the diversity of dietary habits across countries. For instance, dry‐cured Iberian ham is a traditional pork product highly appreciated in Spain due to its culinary qualities, which may contribute to the widespread perception of low risk. However, hepatitis E virus (HEV) has been identified to circulate in Spanish pig populations since decades ago [28, 29], in parallel to increasing trends in the incidence and mortality of HEV infection [30]. In addition, the risk of severe hepatitis A virus (HAV) infection is increased in low or very low endemicity settings, such as Spain, due to the low circulation of HAV and the higher proportion of susceptible adults [26, 31]. The observed behavior involving meat products is noteworthy in view of the occurrence in 2019 of a large listeriosis outbreak due to contaminated stuffed pork in Andalusia, which resulted in more than 200 cases and received extensive media coverage [32].

Although pet ownership is not formally discouraged by the AST‐IDCOP guidelines, emphasis is put on maintaining appropriate veterinary check‐ups, frequent hand washing after handling pets, and avoiding direct contact with animal feces, bird cages or feeders, or ill animals [18]. These recommendations reflect a balance between the well‐recognized risk of transmission of zoonotic pathogens—such as *Bartonella* or *Pasteurella* [33, 34]—and the psychological and social benefits attributed to pet ownership [9]. Our survey shows a good adherence rate to these precautions, similar to previous studies [16]. The high proportion of respondents (≈40%) that reported having considered to relinquish the personal care of their pet suggests the presence of specific concerns that may require targeted health education and counselling. We have found low reported frequencies of gardening and international travel, likely reflecting the relatively low socioeconomic status of the catchment area of our center.

Periodontitis is a highly prevalent condition among ESRD patients that has been shown to contribute to the development of low‐grade systemic inflammation and cardiovascular disease [35]. Poor periodontal status after KT is associated with lower graft and patient survival [36]. In addition, episodes of transient bacteremia during tooth brushing or dental procedures may lead to infective endocarditis [37]. Therefore, maintaining a good oral hygiene with regular referral to a dental clinic is encouraged for SOT recipients [38]. Our survey reveals that the adherence to this recommendation was poor (only 19.7% of participants stated to visit the dentist at least every 3–6 months), and appeared to be impeded by socioeconomic barriers, since patients within the lowest household income tertile were less likely to regularly attend dental visits. The impact of social class inequalities on the prevalence of periodontal disease or the use of oral healthcare services has been reported for the Spanish population [39, 40]. On the other hand, the low frequency of continuous use of sun protection throughout the year is concordant with the rate of SOT recipients that reported the use of sunscreen creams only on sunny days or at vacation time in surveys performed in other countries with high sun exposure [41]. Previous studies in the overall population have observed an inverse association between income status and sun‐protective behaviors [42].

The rates of influenza vaccination uptake were higher than those observed for non‐transplant high‐risk patients with other chronic conditions in our setting [43] or for KT recipients in other countries [44, 45], although the accuracy of self‐reported vaccination status has been found to be moderate at best [46]. More concerning is the low level of knowledge about other vaccines, such as pneumococcal or MMR, as most of the surveyed recipients were not aware of their immunization status. Again, this drawback should justify the implementation of tailored HEP, particularly taking into account the still high incidence rate of vaccine‐preventable infections in SOT recipients [10, 11].

The racial and cultural diversity of patients and healthcare providers has been empirically identified as a factor contributing to lower medication adherence and poor health outcomes [47, 48, 49]. Such an effect is likely extensible to the transplant setting. Previous studies have shown a higher mortality and worse graft survival among US non‐Hispanic black KT recipients [50, 51] or Hispanic patients undergoing hematopoietic stem cell transplantation [52], after controlling for sociodemographic confounding variables. Roma ethnicity has been associated with poorer KT outcomes in some European countries [53, 54]. These findings may be explained by a multiplicity of biological and socioeconomic determinants in addition to differences in the organization and accessibility of healthcare systems, as suggested by the fact that the ethnic origin does not exert an apparent impact on the outcomes of KT recipients in France [55] or the United Kingdom [56]. The Spanish National Healthcare System is a socialized system with universal coverage, in which the transplant activity is predominantly supported by the public sector [57]. In the present experience, 16.9% of the surveyed patients were of foreign origin (mainly from Latin American countries) and 6.2% of them did not have Spanish as their first language. Interestingly, the consumption of raw or undercooked meat and seafood was more commonly acknowledged by this group, which would point to a role for the cultural and ethnic background on food habits and perceptions [58], in addition to potential knowledge gaps and linguistic barriers. In this line, population‐based and case‐control studies carried out in the United States and Australia have reported that listeriosis disproportionately affects ethnic minorities [59, 60] or households in which a language other than English is spoken [61].

Some limitations need to be acknowledged. Beyond the relatively low number of surveyed patients, the questionnaire may have been incorrectly interpreted by some respondents depending on their education level or proficiency in Spanish. We might have directly asked about perceived obstacles for complying with the recommendations, although it would have resulted in a longer and less feasible questionnaire. A further limitation is that we were unable to collect representative information on sexual practices and the risk of sexually transmitted diseases. Although some questions about the sexual domain were included in the survey (e.g. presence of a regular sexual partner, total number of sexual partners in the previous year, or use and type of contraception during the sexual intercourses), these information could not be analyzed due to the low response rate (below 40%). We also lacked data on vaccination in household contacts. Finally, these findings cannot be easily extrapolated to other KT populations with different demographics, sociocultural, or economic backgrounds. On the other hand, the study has some strengths such as the high completion rate of the questionnaire and the comprehensive analysis of numerous domains. The survey was designed to minimize the risk of bias, as some questions unrelated to safe living habits were also included (e.g. HRQoL scales or medication adherence).

In conclusion, most of the surveyed KT recipients showed, already at the early posttransplant period, a reasonable level of knowledge and adherence to recommendations on lifestyle behaviors aimed at reducing immunosuppression‐associated complications. Nevertheless, relevant areas for improvement were also identified concerning food safety, oral hygiene, and sun exposure habits. These results and the evidence of patient‐specific barriers—derived from cultural, educational, and economic disparities—would justify incorporating HEP activities into transplant programs based on dedicated educational materials and counseling. Further studies should investigate the correlation between lifestyle habits and posttransplant outcomes, as well as the potential impact of targeted educational interventions.

## Author Contributions

**Tamara Ruiz‐Merlo**: conceptualization, investigation, methodology, formal analysis, writing – original draft. **Isabel Rodríguez‐Goncer**: data curation, software, investigation. **Francisco López‐Medrano**: supervision, funding acquisition, conceptualization, data curation, software, investigation. **Natalia Polanco**: investigation, software, data curation. **Esther González**: investigation, software, data curation. **Hernando Trujillo**: investigation, software, data curation. **Marina Fayos**: investigation, software, data curation. **Natalia Redondo**: investigation, software, data curation. **Rafael San Juan**: conceptualization, investigation, funding acquisition, software, data curation, supervision. **Amado Andrés**: conceptualization, investigation, data curation. **José María Aguado**: conceptualization, investigation, funding acquisition, software, data curation, supervision, visualization, project administration. **Mario Fernández‐Ruiz**: conceptualization, validation, funding acquisition, writing – review and editing, investigation, resources.

## Conflicts of Interest

The authors declare no conflicts of interest.

## Data Availability

The data that support the findings of this study are available upon reasonable request to the corresponding author.
